# The genome sequence of the Thick-legged Hoverfly, *Syritta pipiens *(Linnaeus, 1758)

**DOI:** 10.12688/wellcomeopenres.19848.1

**Published:** 2023-08-17

**Authors:** Liam M. Crowley, Michael Ashworth, Denise C. Wawman, University of Oxford and Wytham Woods Genome Acquisition Lab, Natural History Museum Genome Acquisition Lab, Darwin Tree of Life Barcoding collective, Wellcome Sanger Institute Tree of Life programme, Wellcome Sanger Institute Scientific Operations: DNA Pipelines collective, Tree of Life Core Informatics collective, Darwin Tree of Life Consortium

**Affiliations:** 1 University of Oxford, Oxford, England, UK; 2 Independent researcher, Yeovil, England, UK

**Keywords:** Syritta pipiens, Thick-legged Hoverfly, genome sequence, chromosomal, Diptera

## Abstract

We present a genome assembly from an individual female *Syritta pipiens* (the Thick-legged Hoverfly; Arthropoda; Insecta; Diptera; Syrphidae). The genome sequence is 318.5 megabases in span. Most of the assembly is scaffolded into 5 chromosomal pseudomolecules. The mitochondrial genome has also been assembled and is 15.76 kilobases in length. Gene annotation of this assembly on Ensembl identified 18,405 protein coding genes.

## Species taxonomy

Eukaryota; Metazoa; Eumetazoa; Bilateria; Protostomia; Ecdysozoa; Panarthropoda; Arthropoda; Mandibulata; Pancrustacea; Hexapoda; Insecta; Dicondylia; Pterygota; Neoptera; Endopterygota; Diptera; Brachycera; Muscomorpha; Eremoneura; Cyclorrhapha; Aschiza; Syrphoidea; Syrphidae; Eristalinae; Xylotini; *Syritta*; *Syritta pipiens* (Linnaeus, 1758) (NCBI:txid34682).

## Background

*Syritta pipiens* is the only representative of this genus of hoverflies in Britain and Ireland. It can be distinguished from other hoverflies in the region by the enlarged hind femora and ash-grey/silverdusting of the lateral thorax ( Ball & Morris, 2015). It is a small, narrow hoverfly with paired orange or grey spots on tergites two and three and a row of on small spines on the ventral surface of the swollen hind femur.

It is widespread and common species, and adults have been recorded in all months of the year visiting a huge variety of flowers ( Ball & Morris, 2015). The larvae are detritivores, feeding on damp decaying vegetable matter such as leaves and compost, but have also been recorded damaging daffodil bulbs ( Hodson, 1931), and feeding on human corpses and thus may have a use in forensic pathology ( Magni *et al.*, 2013). In flight it is an effective mimic of small crabronid wasps.

Males possess large eyes with enlarged anterior facets, which is believed to confer enhanced binocular vision ( Stubbs & Falk, 2002). *This may contribute* to the males’ very efficient visual system for tracking females and remaining 5 to 15 cm away until they are ready to catch the female ( Collett & Land, 1975). This system has inspired a flying robot which chases in a similar manner ( Colonnier *et al.*, 2019).

This is the first full genome sequence to be published for *Syritta pipiens*, but a complete mitochondrial sequence has been published ( Shi *et al.*, 2021). We present a chromosomally complete genome sequence for *S. pipiens*, based on one female specimen from Wytham Woods, as part of the Darwin Tree of Life Project. This project is a collaborative effort to sequence all named eukaryotic species in the Atlantic Archipelago of Britain and Ireland.

## Genome sequence report

**Figure 1.**
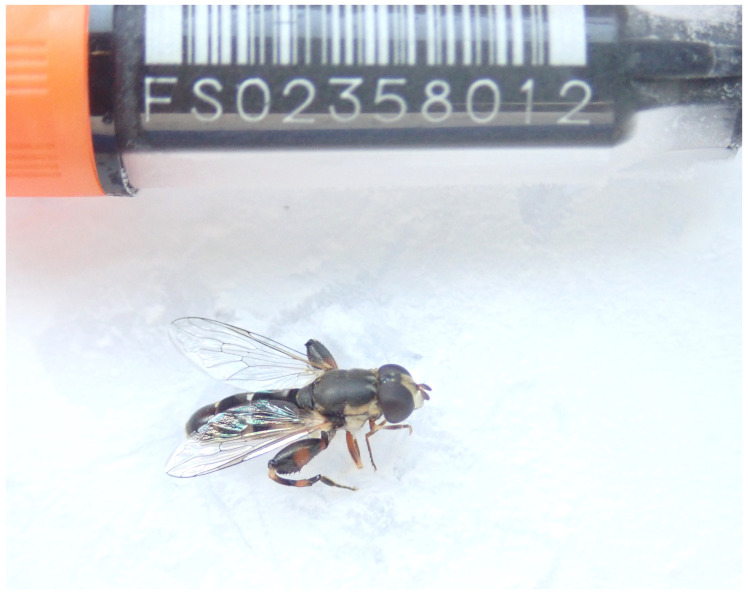
Photograph of the *Syritta pipiens* (idSyrPipi1) specimen used for genome sequencing.

The genome was sequenced from one female *Syritta pipiens* ( Figure 1) collected from Wytham Woods, Oxfordshire (51.77, –1.34). A total of 44-fold coverage in Pacific Biosciences single-molecule HiFi long reads and 123-fold coverage in 10X Genomics read clouds were generated. Primary assembly contigs were scaffolded with chromosome conformation Hi-C data. Manual assembly curation corrected 15 missing joins or mis-joins and removed one haplotypic duplication, reducing the assembly length by 0.95% and the scaffold number by 70%, and increasing the scaffold N50 by 206.63%.

**Figure 2.**
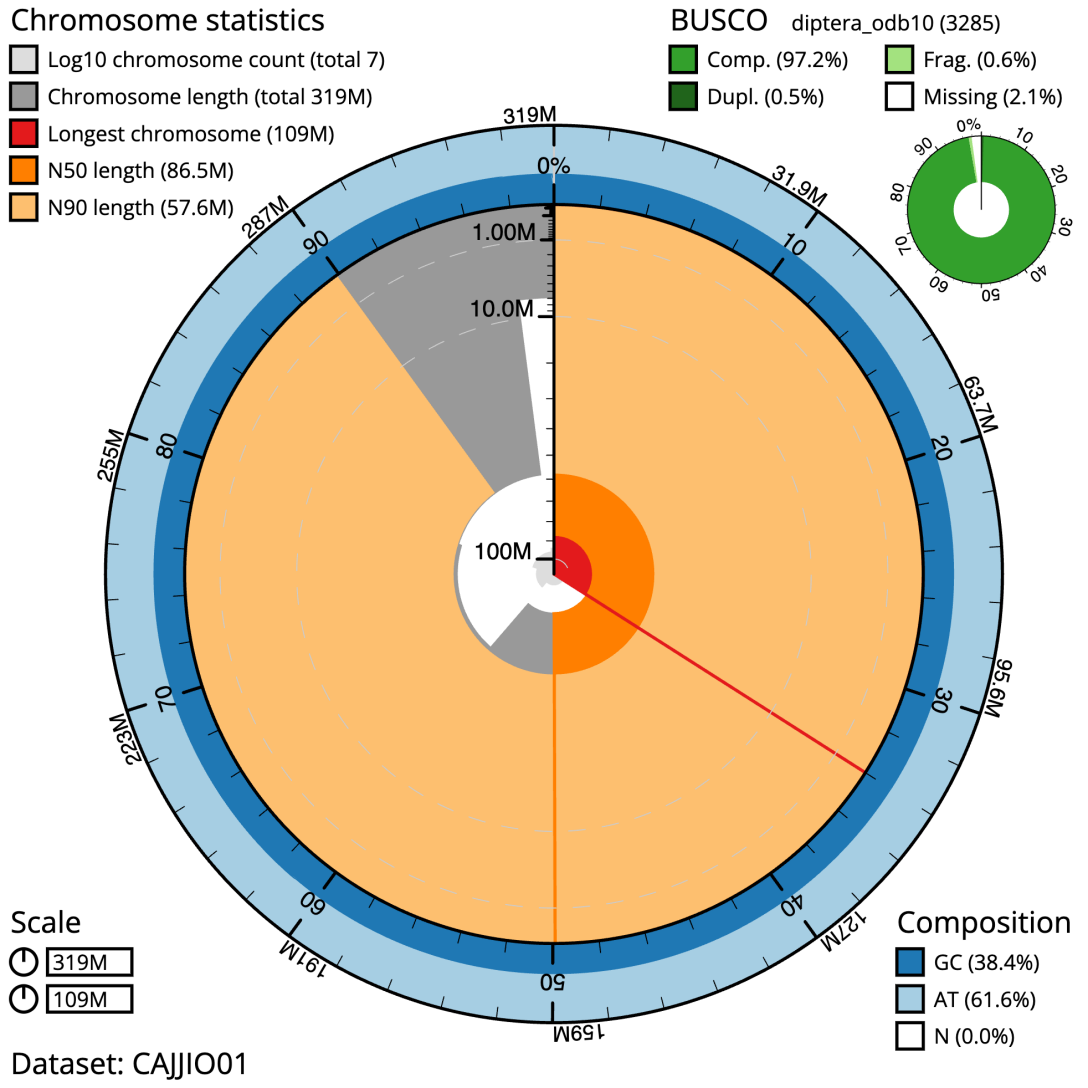
Genome assembly of *Syritta pipiens*, idSyrPipi1.1: metrics. The BlobToolKit Snailplot shows N50 metrics and BUSCO gene completeness. The main plot is divided into 1,000 size-ordered bins around the circumference with each bin representing 0.1% of the 318,522,517 bp assembly. The distribution of sequence lengths is shown in dark grey with the plot radius scaled to the longest sequence present in the assembly (108,597,361 bp, shown in red). Orange and pale-orange arcs show the N50 and N90 sequence lengths (86,509,480 and 57,582,887 bp), respectively. The pale grey spiral shows the cumulative sequence count on a log scale with white scale lines showing successive orders of magnitude. The blue and pale-blue area around the outside of the plot shows the distribution of GC, AT and N percentages in the same bins as the inner plot. A summary of complete, fragmented, duplicated and missing BUSCO genes in the diptera_odb10 set is shown in the top right. An interactive version of this figure is available at https://blobtoolkit.genomehubs.org/view/Syritta pipiens/dataset/CAJJIO01/snail.

The final assembly has a total length of 318.5 Mb in 6 sequence scaffolds with a scaffold N50 of 86.5 Mb ( Table 1). Most (99.98%) of the assembly sequence was assigned to 5 chromosomal-level scaffolds, representing 4 autosomes and the X sex chromosome. Chromosome-scale scaffolds confirmed by the Hi-C data are named in order of size ( Figure 2– Figure 5; Table 2). While not fully phased, the assembly deposited is of one haplotype. Contigs corresponding to the second haplotype have also been deposited. The mitochondrial genome was also assembled and can be found as a contig within the multifasta file of the genome submission.

**Table 1.** Genome data for *Syritta pipiens*, idSyrPipi1.1.

Project accession data		
Assembly identifier	idSyrPipi1.1	
Species	*Syritta pipiens*	
Specimen	idSyrPipi1	
NCBI taxonomy ID	34682	
BioProject	PRJEB42144	
BioSample ID	SAMEA7520166	
Isolate information	idSyrPipi1, female: head and thorax (DNA sequencing and Hi-C scaffolding) idSyrPipi3: thorax (RNA sequencing)	
Assembly metrics *		*Benchmark*
Consensus quality (QV)	55.9	*≥ 50*
*k*-mer completeness	99.99%	*≥ 95%*
BUSCO **	C:97.2%[S:96.7%,D:0.5%], F:0.6%,M:2.1%,n:3,285	*C ≥ 95%*
Percentage of assembly mapped to chromosomes	99.98%	*≥ 95%*
Sex chromosomes	X chromosome	*localised homologous pairs*
Organelles	Mitochondrial genome assembled	*complete single alleles*
Raw data accessions		
PacificBiosciences SEQUEL II	ERR6608651	
10X Genomics Illumina	ERR6002574, ERR6002576, ERR6002577, ERR6002575	
Hi-C Illumina	ERR6003035	
PolyA RNA-Seq Illumina	ERR9434965	
Genome assembly		
Assembly accession	GCA_905187475.1	
*Accession of alternate haplotype*	GCA_905147025.1	
Span (Mb)	318.5	
Number of contigs	23	
Contig N50 length (Mb)	28.2	
Number of scaffolds	6	
Scaffold N50 length (Mb)	86.5	
Longest scaffold (Mb)	108.6	
Genome annotation		
Number of protein-coding genes	11,749	
Number of non-coding genes	1,293	
Number of gene transcripts	18,405	

* Assembly metric benchmarks are adapted from column VGP-2020 of “Table 1: Proposed standards and metrics for defining genome assembly quality” from ( Rhie *et al.*, 2021). ** BUSCO scores based on the diptera_odb10 BUSCO set using v5.3.2. C = complete [S = single copy, D = duplicated], F = fragmented, M = missing, n = number of orthologues in comparison. A full set of BUSCO scores is available at https://blobtoolkit.genomehubs.org/view/Syritta pipiens/dataset/CAJJIO01/busco.

**Figure 3.**
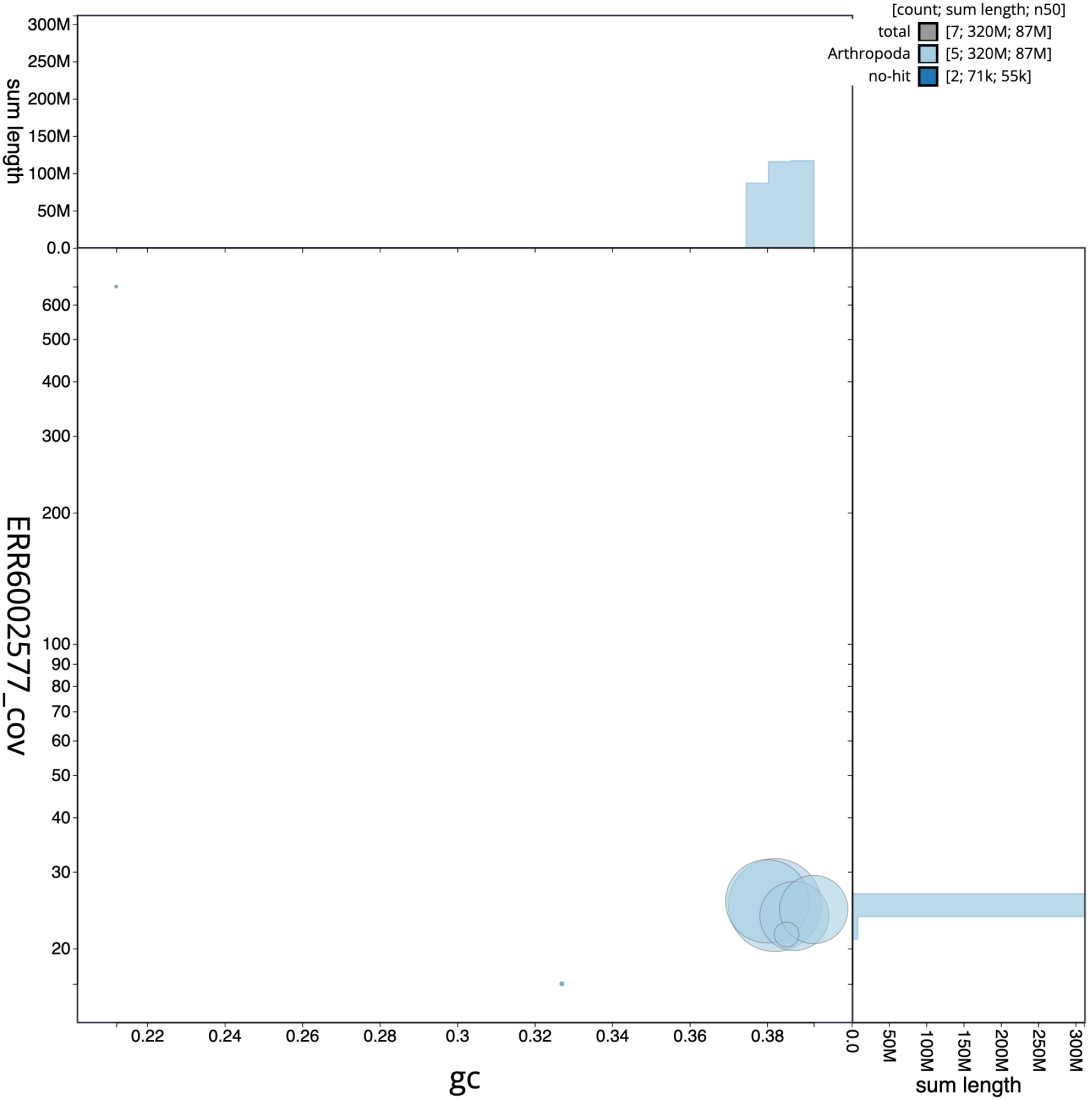
Genome assembly of *Syritta pipiens*, idSyrPipi1.1: BlobToolKit GC-coverage plot. Scaffolds are coloured by phylum. Circles are sized in proportion to scaffold length. Histograms show the distribution of scaffold length sum along each axis. An interactive version of this figure is available at https://blobtoolkit.genomehubs.org/view/Syritta%20pipiens/dataset/CAJJIO01/blob.

**Figure 4.**
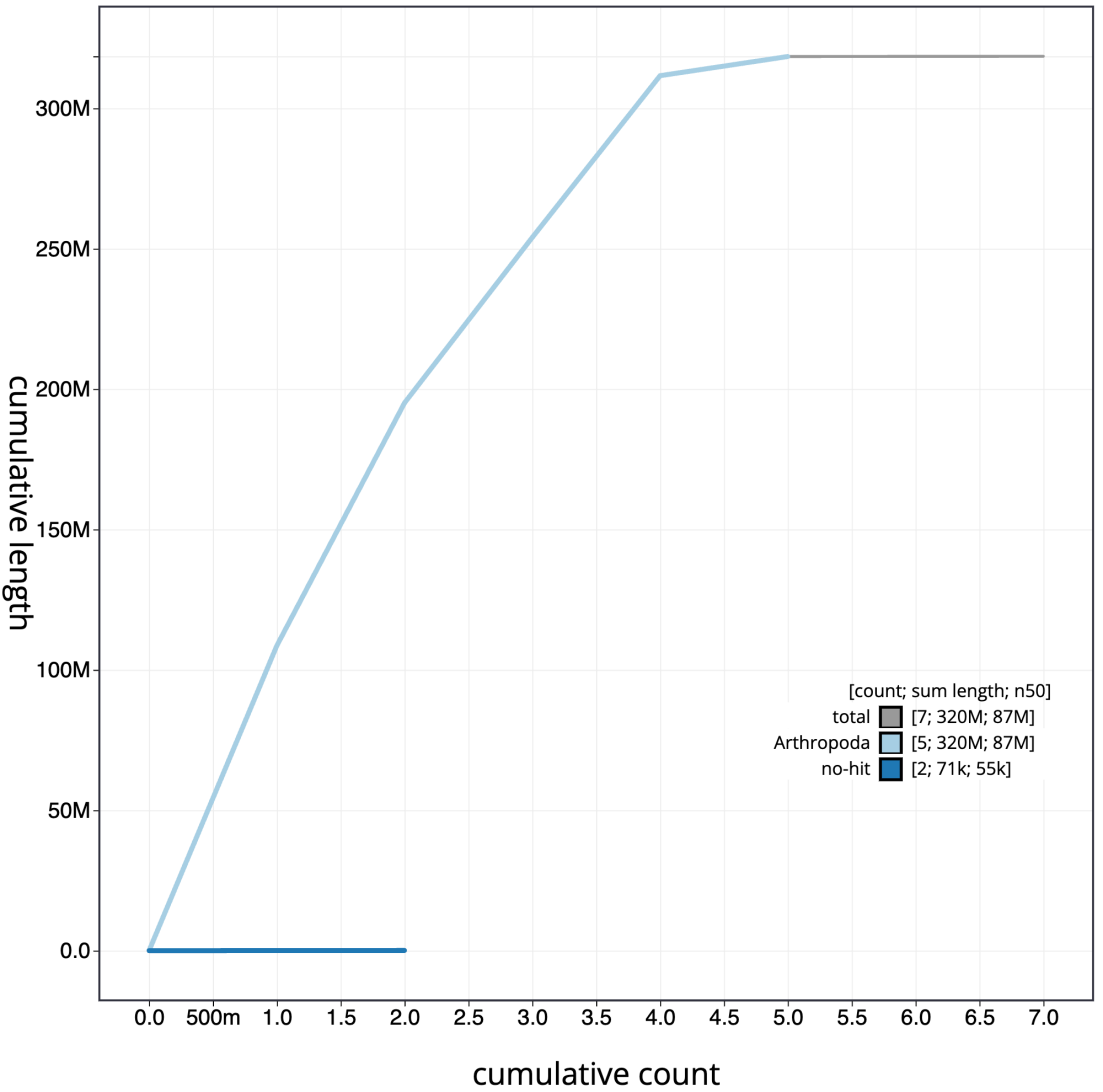
Genome assembly of *Syritta pipiens*, idSyrPipi1.1: BlobToolKit cumulative sequence plot. The grey line shows cumulative length for all scaffolds. Coloured lines show cumulative lengths of scaffolds assigned to each phylum using the buscogenes taxrule. An interactive version of this figure is available at https://blobtoolkit.genomehubs.org/view/Syritta%20pipiens/dataset/CAJJIO01/cumulative.

**Figure 5.**
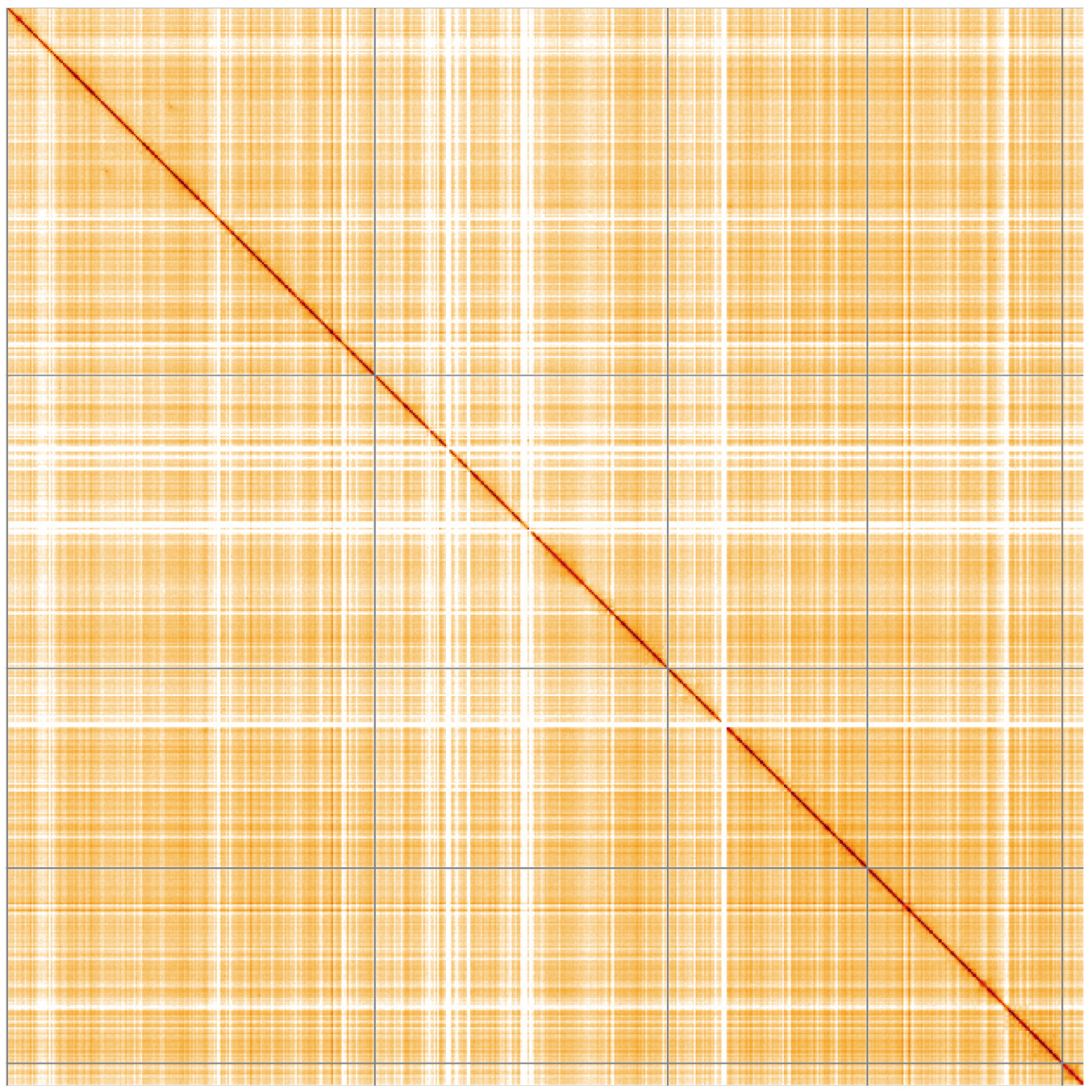
Genome assembly of *Syritta pipiens*, idSyrPipi1.1: Hi-C contact map of the idSyrPipi1.1 assembly, visualised using HiGlass. Chromosomes are shown in order of size from left to right and top to bottom. An interactive version of this figure may be viewed at https://genome-note-higlass.tol.sanger.ac.uk/l/?d=WbDLb3N3SHuZ-PBlaziUYA.

**Table 2.** Chromosomal pseudomolecules in the genome assembly of *Syritta pipiens*, idSyrPipi1.

INSDC accession	Chromosome	Length (Mb)	GC%
LR994571.1	1	108.6	38.0
LR994572.1	2	86.51	38.0
LR994573.1	3	58.93	38.5
LR994574.1	4	57.58	39.0
LR994575.1	X	6.83	38.5
LR994576.1	MT	0.02	21.0

The estimated Quality Value (QV) of the final assembly is 55.9 with *k*-mer completeness of 99.99%, and the assembly has a BUSCO v5.3.2 completeness of 97.2% (single = 96.7%, duplicated = 0.5%), using the diptera_odb10 reference set ( *n* = 3,285).

Metadata for specimens, spectral estimates, sequencing runs, contaminants and pre-curation assembly statistics can be found at https://links.tol.sanger.ac.uk/species/34682.

## Genome annotation report

The *Syritta pipiens* genome assembly (GCA_905187475.1) was annotated using the Ensembl rapid annotation pipeline ( Table 1; https://rapid.ensembl.org/Syritta_pipiens_GCA_905187475.1/Info/Index). The resulting annotation includes 18,405 transcribed mRNAs from 11,749 protein-coding and 1,293 non-coding genes.

## Methods

### Sample acquisition and nucleic acid extraction

A female *Syritta pipiens* (specimen ID Ox000241, ToLID idSyrPipi1) was collected from rough Common in Wytham Woods, Oxfordshire (biological vice-county Berkshire), UK (latitude 51.77, longitude –1.34) on 2019-09-03 by netting. The specimen was collected and identified by Liam Crowley (University of Oxford) and preserved on dry ice.

The specimen used for RNA sequencing (specimen ID NHMUK014111601, ToLID idSyrPipi3) was collected from Orchard House, England (50.97, –2.67) by netting on 2020-07-23. The specimen was collected and identified by Michael Ashworth for the Natural History Museum. The specimen was preserved in liquid nitrogen.

DNA was extracted at the Tree of Life laboratory, Wellcome Sanger Institute (WSI). The idSyrPipi1 sample was weighed and dissected on dry ice with tissue set aside for Hi-C sequencing. Head and thorax tissue was disrupted using a Nippi Powermasher fitted with a BioMasher pestle. High molecular weight (HMW) DNA was extracted using the Qiagen MagAttract HMW DNA extraction kit. Low molecular weight DNA was removed from a 20 ng aliquot of extracted DNA using the 0.8X AMpure XP purification kit prior to 10X Chromium sequencing; a minimum of 50 ng DNA was submitted for 10X sequencing. HMW DNA was sheared into an average fragment size of 12–20 kb in a Megaruptor 3 system with speed setting 30. Sheared DNA was purified by solid-phase reversible immobilisation using AMPure PB beads with a 1.8X ratio of beads to sample to remove the shorter fragments and concentrate the DNA sample. The concentration of the sheared and purified DNA was assessed using a Nanodrop spectrophotometer and Qubit Fluorometer and Qubit dsDNA High Sensitivity Assay kit. Fragment size distribution was evaluated by running the sample on the FemtoPulse system.

RNA was extracted from thorax tissue of idSyrPipi3 in the Tree of Life Laboratory at the WSI using TRIzol, according to the manufacturer’s instructions. RNA was then eluted in 50 μl RNAse-free water and its concentration assessed using a Nanodrop spectrophotometer and Qubit Fluorometer using the Qubit RNA Broad-Range (BR) Assay kit. Analysis of the integrity of the RNA was done using Agilent RNA 6000 Pico Kit and Eukaryotic Total RNA assay.

### Sequencing

Pacific Biosciences HiFi circular consensus and 10X Genomics read cloud DNA sequencing libraries were constructed according to the manufacturers’ instructions. Poly(A) RNA-Seq libraries were constructed using the NEB Ultra II RNA Library Prep kit. DNA and RNA sequencing were performed by the Scientific Operations core at the WSI on Pacific Biosciences SEQUEL II (HiFi), Illumina HiSeq 4000 (RNA-Seq) and HiSeq X Ten (10X) instruments. Hi-C data were also generated from idSyrPipi1 using the Qiagen kit and sequenced on the HiSeq X Ten instrument.

### Genome assembly, curation and evaluation

Assembly was carried out with Hifiasm ( Cheng *et al.*, 2021) and haplotypic duplication was identified and removed with purge_dups ( Guan *et al.*, 2020). One round of polishing was performed by aligning 10X Genomics read data to the assembly with Long Ranger ALIGN, calling variants with FreeBayes ( Garrison & Marth, 2012). The assembly was then scaffolded with Hi-C data ( Rao *et al.*, 2014) using SALSA2 ( Ghurye *et al.*, 2019). The assembly was checked for contamination and corrected using the gEVAL system ( Chow *et al.*, 2016) as described previously ( Howe *et al.*, 2021). Manual curation was performed using gEVAL, HiGlass ( Kerpedjiev *et al.*, 2018) and Pretext ( Harry, 2022). The mitochondrial genome was assembled using MitoHiFi ( Uliano-Silva *et al.*, 2023), which runs MitoFinder ( Allio *et al.*, 2020) or MITOS ( Bernt *et al.*, 2013) and uses these annotations to select the final mitochondrial contig and to ensure the general quality of the sequence.

A Hi-C map for the final assembly was produced using bwa-mem2 ( Vasimuddin *et al.*, 2019) in the Cooler file format ( Abdennur & Mirny, 2020). To assess the assembly metrics, the *k*-mer completeness and QV consensus quality values were calculated in Merqury ( Rhie *et al.*, 2020). This work was done using Nextflow ( Di Tommaso *et al.*, 2017) DSL2 pipelines “sanger-tol/readmapping” ( Surana *et al.*, 2023a) and “sanger-tol/genomenote” ( Surana *et al.*, 2023b). The genome was analysed within the BlobToolKit environment ( Challis *et al.*, 2020) and BUSCO scores ( Manni *et al.*, 2021; Simão *et al.*, 2015) were calculated.

Table 3 contains a list of relevant software tool versions and sources.

**Table 3.** Software tools: versions and sources.

Software tool	Version	Source
BlobToolKit	4.1.7	https://github.com/blobtoolkit/blobtoolkit
BUSCO	5.3.2	https://gitlab.com/ezlab/busco
FreeBayes	1.3.1-17-gaa2ace8	https://github.com/freebayes/freebayes
gEVAL	N/A	https://geval.org.uk/
Hicanu	1.0	https://github.com/marbl/canu
HiGlass	1.11.6	https://github.com/higlass/higlass
Long Ranger ALIGN	2.2.2	https://support.10xgenomics.com/genome-exome/software/pipelines/latest/advanced/other-pipelines
Merqury	MerquryFK	https://github.com/thegenemyers/MERQURY.FK
MitoHiFi	2	https://github.com/marcelauliano/MitoHiFi
PretextView	0.2	https://github.com/wtsi-hpag/PretextView
purge_dups	1.2.3	https://github.com/dfguan/purge_dups
SALSA	2.2	https://github.com/salsa-rs/salsa
sanger-tol/genomenote	v1.0	https://github.com/sanger-tol/genomenote
sanger-tol/readmapping	1.1.0	https://github.com/sanger-tol/readmapping/tree/1.1.0

### Genome annotation

The Ensembl gene annotation system ( Aken *et al.*, 2016) was used to generate annotation for the *Syritta pipiens* assembly (GCA_905187475.1). Annotation was created primarily through alignment of transcriptomic data to the genome, with gap filling via protein-to-genome alignments of a select set of proteins from UniProt ( UniProt Consortium, 2019).

### Wellcome Sanger Institute – Legal and Governance

The materials that have contributed to this genome note have been supplied by a Darwin Tree of Life Partner. The submission of materials by a Darwin Tree of Life Partner is subject to the **‘Darwin Tree of Life Project Sampling Code of Practice’**, which can be found in full on the Darwin Tree of Life website here. By agreeing with and signing up to the Sampling Code of Practice, the Darwin Tree of Life Partner agrees they will meet the legal and ethical requirements and standards set out within this document in respect of all samples acquired for, and supplied to, the Darwin Tree of Life Project.

Further, the Wellcome Sanger Institute employs a process whereby due diligence is carried out proportionate to the nature of the materials themselves, and the circumstances under which they have been/are to be collected and provided for use. The purpose of this is to address and mitigate any potential legal and/or ethical implications of receipt and use of the materials as part of the research project, and to ensure that in doing so we align with best practice wherever possible. The overarching areas of consideration are:

Ethical review of provenance and sourcing of the material

Legality of collection, transfer and use (national and international) 

Each transfer of samples is further undertaken according to a Research Collaboration Agreement or Material Transfer Agreement entered into by the Darwin Tree of Life Partner, Genome Research Limited (operating as the Wellcome Sanger Institute), and in some circumstances other Darwin Tree of Life collaborators.

## Data Availability

European Nucleotide Archive: *Syritta pipiens* (thick-legged hoverfly). Accession number PRJEB42144; https://identifiers.org/ena.embl/PRJEB42144. ( Wellcome Sanger Institute, 2021) The genome sequence is released openly for reuse. The *Syritta pipiens* genome sequencing initiative is part of the Darwin Tree of Life (DToL) project. All raw sequence data and the assembly have been deposited in INSDC databases. Raw data and assembly accession identifiers are reported in Table 1.

## Author information

Members of the University of Oxford and Wytham Woods Genome Acquisition Lab are listed here: https://doi.org/10.5281/zenodo.4789928.

Members of the Natural History Museum Genome Acquisition Lab are listed here: https://doi.org/10.5281/zenodo.4790042.

Members of the Darwin Tree of Life Barcoding collective are listed here: https://doi.org/10.5281/zenodo.4893703.

Members of the Wellcome Sanger Institute Tree of Life programme are listed here: https://doi.org/10.5281/zenodo.4783585.

Members of Wellcome Sanger Institute Scientific Operations: DNA Pipelines collective are listed here: https://doi.org/10.5281/zenodo.4790455.

Members of the Tree of Life Core Informatics collective are listed here: https://doi.org/10.5281/zenodo.5013541.

Members of the Darwin Tree of Life Consortium are listed here: https://doi.org/10.5281/zenodo.4783558.

## References

[ref-1] Abdennur N Mirny LA : Cooler: Scalable storage for Hi-C data and other genomically labeled arrays. *Bioinformatics.* 2020;36(1):311–316. 10.1093/bioinformatics/btz540 31290943 PMC8205516

[ref-2] Aken BL Ayling S Barrell D : The Ensembl gene annotation system. *Database (Oxford).* 2016;2016: baw093. 10.1093/database/baw093 27337980 PMC4919035

[ref-3] Allio R Schomaker‐Bastos A Romiguier J : MitoFinder: Efficient automated large‐scale extraction of mitogenomic data in target enrichment phylogenomics. *Mol Ecol Resour.* 2020;20(4):892–905. 10.1111/1755-0998.13160 32243090 PMC7497042

[ref-4] Ball SG Morris R : Syritta pipiens.In: *Britain’s Hoverflies: A Field Guide - Revised and Updated Second Edition.* Princeton University Press,2015;266–267.

[ref-5] Bernt M Donath A Jühling F : MITOS: Improved *de novo* metazoan mitochondrial genome annotation. *Mol Phylogenet Evol.* 2013;69(2):313–319. 10.1016/j.ympev.2012.08.023 22982435

[ref-6] Challis R Richards E Rajan J : BlobToolKit - interactive quality assessment of genome assemblies. *G3 (Bethesda).* 2020;10(4):1361–1374. 10.1534/g3.119.400908 32071071 PMC7144090

[ref-7] Cheng H Concepcion GT Feng X : Haplotype-resolved *de novo* assembly using phased assembly graphs with hifiasm. *Nat Methods.* 2021;18(2):170–175. 10.1038/s41592-020-01056-5 33526886 PMC7961889

[ref-8] Chow W Brugger K Caccamo M : gEVAL — a web-based browser for evaluating genome assemblies. *Bioinformatics.* 2016;32(16):2508–2510. 10.1093/bioinformatics/btw159 27153597 PMC4978925

[ref-9] Collett TS Land MF : Visual control of flight behaviour in the hoverfly *Syritta pipiens* L. *J Comp Physiol.* 1975;99(1):1–66. 10.1007/BF01464710

[ref-10] Colonnier F Ramirez-Martinez S Viollet S : A bio-inspired sighted robot chases like a hoverfly. *Bioinspir Biomim.* 2019;14(3): 036002. 10.1088/1748-3190/aaffa4 30654332

[ref-11] Di Tommaso P Chatzou M Floden EW : Nextflow enables reproducible computational workflows. *Nat Biotechnol.* 2017;35(4):316–319. 10.1038/nbt.3820 28398311

[ref-12] Garrison E Marth G : Haplotype-based variant detection from short-read sequencing.2012. 10.48550/arXiv.1207.3907

[ref-13] Ghurye J Rhie A Walenz BP : Integrating Hi-C links with assembly graphs for chromosome-scale assembly. *PLoS Comput Biol.* 2019;15(8): e1007273. 10.1371/journal.pcbi.1007273 31433799 PMC6719893

[ref-14] Guan D McCarthy SA Wood J : Identifying and removing haplotypic duplication in primary genome assemblies. *Bioinformatics.* 2020;36(9):2896–2898. 10.1093/bioinformatics/btaa025 31971576 PMC7203741

[ref-15] Harry E : PretextView (Paired REad TEXTure Viewer): A desktop application for viewing pretext contact maps.2022; [Accessed 19 October 2022]. Reference Source

[ref-16] Hodson WEH : A comparison of the immature Stages of *Eumerus tuberculatus*, Rond., and *Syritta pipiens*, Linn. (Syrphidae). *Bull Entomol Res.* 1931;22(1):55–58. 10.1017/S0007485300029734

[ref-17] Howe K Chow W Collins J : Significantly improving the quality of genome assemblies through curation. *GigaScience.* Oxford University Press,2021;10(1): giaa153. 10.1093/gigascience/giaa153 33420778 PMC7794651

[ref-18] Kerpedjiev P Abdennur N Lekschas F : HiGlass: web-based visual exploration and analysis of genome interaction maps. *Genome Biol.* 2018;19(1): 125. 10.1186/s13059-018-1486-1 30143029 PMC6109259

[ref-19] Magni PA Pérez-Bañón C Borrini M : *Syritta pipiens* (Diptera: Syrphidae), a new species associated with human cadavers. *Forensic Sci Int.* 2013;231(1–3):e19–e23. 10.1016/j.forsciint.2013.05.023 23806343

[ref-20] Manni M Berkeley MR Seppey M : BUSCO update: Novel and streamlined workflows along with broader and deeper phylogenetic coverage for scoring of eukaryotic, prokaryotic, and viral genomes. *Mol Biol Evol.* 2021;38(10):4647–4654. 10.1093/molbev/msab199 34320186 PMC8476166

[ref-21] Rao SSP Huntley MH Durand NC : A 3D map of the human genome at kilobase resolution reveals principles of chromatin looping. *Cell.* 2014;159(7):1665–1680. 10.1016/j.cell.2014.11.021 25497547 PMC5635824

[ref-22] Rhie A McCarthy SA Fedrigo O : Towards complete and error-free genome assemblies of all vertebrate species. *Nature.* 2021;592(7856):737–746. 10.1038/s41586-021-03451-0 33911273 PMC8081667

[ref-23] Rhie A Walenz BP Koren S : Merqury: Reference-free quality, completeness, and phasing assessment for genome assemblies. *Genome Biol.* 2020;21(1): 245. 10.1186/s13059-020-02134-9 32928274 PMC7488777

[ref-24] Shi YQ Li J Li H : The complete mitochondrial genome of *Syritta pipiens* (Linnaeus, 1758) (Diptera: Syrphidae) and phylogenetic analysis. *Mitochondrial DNA B Resour.* 2021;6(9):2475–2477. 10.1080/23802359.2021.1957035 34368448 PMC8317924

[ref-25] Simão FA Waterhouse RM Ioannidis P : BUSCO: assessing genome assembly and annotation completeness with single-copy orthologs. *Bioinformatics.* 2015;31(19):3210–2. 10.1093/bioinformatics/btv351 26059717

[ref-26] Stubbs AE Falk SJ : British hoverflies: an illustrated identification guide.British Entomological and Natural History Society,2002.

[ref-27] Surana P Muffato M Qi G : sanger-tol/readmapping: sanger-tol/readmapping v1.1.0 - Hebridean Black (1.1.0). *Zenodo.* 2023a. 10.5281/zenodo.7755665

[ref-28] Surana P Muffato M Sadasivan Baby C : sanger-tol/genomenote (v1.0.dev). *Zenodo.* 2023b. Reference Source

[ref-29] Uliano-Silva M Ferreira JGRN Krasheninnikova K : MitoHiFi: a python pipeline for mitochondrial genome assembly from PacBio high fidelity reads. *BMC Bioinformatics.* 2023;24(1): 288. 10.1186/s12859-023-05385-y 37464285 PMC10354987

[ref-30] UniProt Consortium: UniProt: a worldwide hub of protein knowledge. *Nucleic Acids Res.* 2019;47(D1):D506–D515. 10.1093/nar/gky1049 30395287 PMC6323992

[ref-31] Vasimuddin M Misra S Li H : Efficient Architecture-Aware Acceleration of BWA-MEM for Multicore Systems.In: *2019 IEEE International Parallel and Distributed Processing Symposium (IPDPS).*IEEE,2019;314–324. 10.1109/IPDPS.2019.00041

[ref-32] Wellcome Sanger Institute: The genome sequence of the Thick-legged Hoverfly, *Syritta pipiens* (Linnaeus, 1758). European Nucleotide Archive.[dataset], accession number PRJEB42144,2021.

