# Supplementary material for: The genome sequence of the Thick-legged Hoverfly, Syritta pipiens (Linnaeus, 1758)
Source: Wellcome Open Res. 2023 Aug 17;8:349. [Version 1] doi: 10.12688/wellcomeopenres.19848.1 (PMC11303951; doi:10.12688/wellcomeopenres.19848.1)
Supplement: Peer review history

# Reviewer report: Reviewer response for version 1

**DOI:** 10.21956/wellcomeopenres.21981.r88669

**Published:** 2024-08-09

**Authors:** Muzafar Riyaz

**Affiliations:** 1 Xavier Research Foundation, St Xavier's College (Ringgold ID: 29983), Palayamkottai, Tamil Nadu, India

The genome assembly of *Syritta pipiens* is a valuable addition to the Darwin Tree of Life Project, providing a comprehensive genetic resource for future research. The data note effectively presents the genome sequence, assembly metrics, and gene annotation, demonstrating thoroughness in data collection and processing. However, a few aspects could enhance the utility and clarity of the data note:

- **Data Accessibility:** The inclusion of direct links to the interactive visualizations and raw data repositories, such as BlobToolKit and European Nucleotide Archive, is commendable. To further improve accessibility, consider providing a step-by-step guide or a brief tutorial on how to navigate and utilize these resources effectively.

- **Detailed Metadata:** While the data note includes metadata for the specimen and sequencing runs, more detailed information about the environmental conditions and specific collection methods could be beneficial for researchers attempting to replicate or build upon this study.
- **Quality Control Metrics:** The assembly quality metrics (e.g., N50, BUSCO scores) are well-documented, but a comparative table listing these metrics alongside those from related species could provide additional context for evaluating the assembly's quality.
- **Future Research Directions:** Although the primary focus is on data presentation, a brief section suggesting potential research applications and implications of this genome assembly could guide researchers in leveraging this data for various scientific inquiries.

Overall, the data note is well-structured and provides a comprehensive overview of the genome assembly for *Syritta pipiens*. Addressing the suggestions above could further enhance its clarity, accessibility, and utility for the research community.

Are sufficient details of methods and materials provided to allow replication by others?

Yes

Is the rationale for creating the dataset(s) clearly described?

Yes

Are the datasets clearly presented in a useable and accessible format?

Yes

Are the protocols appropriate and is the work technically sound?

Yes

Reviewer Expertise:

Genomics, Phylogenetics, Phylogenomics, Next Generation sequencing

I confirm that I have read this submission and believe that I have an appropriate level of expertise to confirm that it is of an acceptable scientific standard.

# Reviewer report: Reviewer response for version 1

**DOI:** 10.21956/wellcomeopenres.21981.r88663

**Published:** 2024-08-06

**Authors:** Annabel Whibley

**Affiliations:** 1 The University of Auckland, Auckland, Auckland, New Zealand

Crowley, Ashworth, Wawman and colleagues present a reference genome assembly and annotation of the Thick-legged Hoverfly ( *Syritta pipiens*). The reference is high-quality, and has been constructed using appropriate tools and with comprehensive reporting of the sample collection, data generation and analysis and all associated metadata. Links to data accessions are functional.

 That the gene annotation was informed by RNAseq is worth highlighting, perhaps even in the abstract.

 A comment on the sequence identity of the published mtDNA sequence to your assembled one here would be good to include.

**Minor comments:**

- Typo in background, “It is a widespread and common species…”
- Lingering italics in *“This may contribute* to the males’ very efficient visual system for …”
- “Rough” should be capitalised in “A female *Syritta pipiens* (specimen ID Ox000241, ToLID idSyrPipi1) was collected from rough Common in Wytham Woods,” 
- I will continue to query whether this templated detail is correct: “in brief, the method employs a 1.8X ratio of AMPure PB beads to sample to eliminate shorter fragments and concentrate the DNA.”  This ratio of beads is not size-selective, I believe this should be 0.6x, as in the protocols.io Guidelines.

Are sufficient details of methods and materials provided to allow replication by others?

Yes

Is the rationale for creating the dataset(s) clearly described?

Yes

Are the datasets clearly presented in a useable and accessible format?

Yes

Are the protocols appropriate and is the work technically sound?

Yes

Reviewer Expertise:

Genomics, Bioinformatics, Evolution

I confirm that I have read this submission and believe that I have an appropriate level of expertise to confirm that it is of an acceptable scientific standard.

# Reviewer report: Reviewer response for version 1

**DOI:** 10.21956/wellcomeopenres.21981.r65454

**Published:** 2023-09-06

**Authors:** Kevin M Moran

**Affiliations:** 1 Canadian National Collection of Insects Arachnids and Nematodes, Ottawa, Ontario, Canada

The authors provide a chromosome-level assembly of the *Syritta pipiens* genome. The metholodogy including the assembly of PacBio HiFi reads, assembly polishing and scaffoldin is appropriate, clearly described and reproducible. The reasons for investigation are sound.

 How did you verify that this specimen represents *Syritta pipiens?* It is unlikely, yet possible, that additional species of *Syritta* may be introduced and ultimately establish. 

 I recommend including at least the COI barcode of the specimen in the paper. This would validate identification and may solve future headaches if a taxonomist later finds a species is a complex.

Are sufficient details of methods and materials provided to allow replication by others?

Yes

Is the rationale for creating the dataset(s) clearly described?

Yes

Are the datasets clearly presented in a useable and accessible format?

Yes

Are the protocols appropriate and is the work technically sound?

Yes

Reviewer Expertise:

Taxonomy, Phylogenetics, Bioinformatics.

I confirm that I have read this submission and believe that I have an appropriate level of expertise to confirm that it is of an acceptable scientific standard.
